# The effect of climatic and geographical factors on breast cancer in Iran

**DOI:** 10.1186/s13104-020-05368-9

**Published:** 2020-11-10

**Authors:** Zohreh Maryanaji

**Affiliations:** Department of Geography, Sayyed Jamaleddin Asadabadi University, 6541835583 Asadabad, Iran

**Keywords:** Solar radiation, Breast cancer, Ultraviolet radiation, Climatic factors

## Abstract

**Objective:**

By studying the effect of environmental factors on health, it is clear that geographical, climatic and environmental factors have a significant impact on human health. This study, based on the data of the patients with breast cancer in Iran since 2010 to 2014 and using the statistical methods has determined the effect of geographical features of Iran (solar radiation status, radiation angle) on the frequency and distribution of this disease.

**Results:**

The maximum amount of total solar radiation occurs in the vicinity (surrounding) of the tropic of cancer, which covers some parts of the south of Iran and in the atmosphere of the northern latitudes of Iran. The amount of humidity and cloudiness is more than the southern latitudes, which causes more reflection of short waves of the sun during the day. Findings showed that the rate of breast cancer in low latitudes is higher than high latitudes. It was also found that with increasing longitude, the rate of cancer increases significantly due to the high thickness of the atmosphere and receiving more sunlight in the electromagnetic spectrum, as well as dry air and low water vapor in low altitude areas of eastern and southeastern Iran.

## Introduction

Environmental and climatic conditions in each region cause the occurrence and spread of more diseases [20, 21]. Medical knowledge uses geographical research in its study and can identify the relationship between disease and environmental conditions through disease patterns in geographical areas. Cancer is also one of the diseases influenced by environmental factors. Prevalence of cancer varies in different geographical areas.

Iran has the highest cancer growth in the world and it is predicted to be the second cause of mortality in Iran by 2025. Breast cancer is one of the most common and deadly cancer in Iranian women [1].

Table 1 shows the rate of patients with cancer in Iran in 2013.

**Table 1:** The frequency of various types of cancer in 2013

Type of cancer	The percentage of patients	The number of patients (person)
Skin	21.5	6.046
Stomach	15.5	4.254
Breast	14.5	3.946
Bladder	10.1	2.775
Cologne	10	2.759
Gullet	8.5	2.339
Blood	6.7	1.734
Prostate	5.5	1.548
Lymph nodes	4.5	1.255
Lung	3.2	873
Sum	100	27.529

Breast cancer is a disease that occurs when some breast cells begin to grow abnormally. Researchers have found that hormonal, lifestyle and environmental factors may increase the risk of breast cancer. It is also possible that breast cancer is caused by a complex interaction of genetic and environmental factors. Many researchers have studied the role of environmental and geographical factors in the incidence of this disease, some of which are listed below. In the study of the risk of breast cancer, considering the time spent in the sun, it was found that being more than one hour a day in the sun in summer can reduce the risk of breast cancer [13]. The impact of environment on breast cancer shows that exposure to the environment along with genetic background, age, and hormonal factors have an overall impact on breast cancer risk [7]. These ecological studies, despite their limitations, generally support the hypothesis that exposure to the sun's ultraviolet rays may play a significant role in the risk of breast cancer [2, 4, 11, 12, 15, 18].

Studies have also been conducted on women's health and resilience to climate change and natural and environmental disasters [5, 19–21].

In industrialized societies, different environments due to different solar radiation have led to endocrine disorders and thus increased the risk of breast cancer [22]. Pooled analysis and the latest meta-analysis showed that exposure to the sun's ultraviolet rays reduced the risk of developing the disease by increasing vitamin D levels [8, 16]. A study of the risk of cancer mortality in high latitude geographical areas of Japan has shown that increased exposure to sunlight reduces the risk of various types of cancer [17]. Differences in geographical location may result in heterogeneity of results between menopausal subgroups and hormone receptors [3]. Differences in the amount of UV exposure in the United States may be the cause of significant regional differences in breast cancer mortality [10].

In addition, there is evidence that exposure to ultraviolet rays during the periods of childhood and adolescence may be directly related to the incidence of breast cancer [6, 23]. Other studies have found that cancer mortality for all invasive cancers is not significantly associated with increased sunlight, but for 7 of the 22 precursor cancers, death is predicted by increased solar radiation [9].

There are two ways to look at exposure to the sun's ultraviolet ray and their association with breast cancer: (1). ultraviolet radiation, which is an ecological measure and is not practical in large studies. (2). Time spent in the sun—the numbers of hours a person spends on average on weekends outside the home- is determined. Evaluation results of these two cases were in conflict with each other [24].

The incidence of cancers in any region depends on racial, geographical and environmental conditions. The aim of this study was to identify the geographical distribution of breast cancer prevalence in Iran and its prevalence in different geographical areas. Given the importance of breast cancer in Iran, we considered those geographical parameters whose roles in cancer have been less studied by others or have not yet been evaluated. Therefore, the effect of latitude and longitude directly (irradiation angle) and indirectly (day length) on the incidence or absence of breast cancer in arid and semi-arid climate of Iran has been investigated. This study has analyzed the statistical population of patients with breast cancer in Iran from 2010 to 2014.

## Main text

### Material and methods

#### Study area

Between 25° and 39° north latitude and 44° to 63° east longitude.

**Fig. 1:**
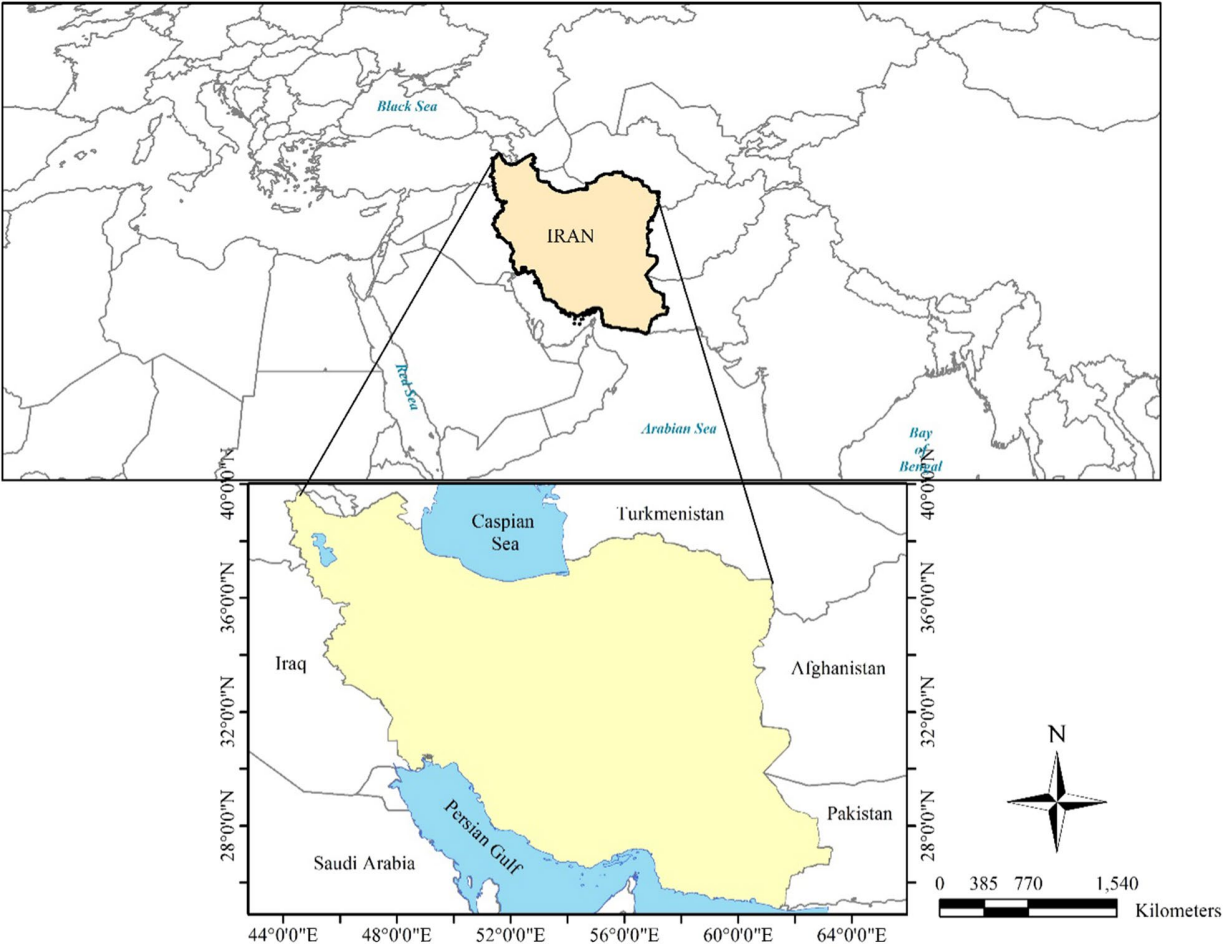
Geographical location of Iran (It was drawn by Z. Maryanaji using free QGIS software)

Iran lays on the northern hemisphere between 25° and 40° N latitudes and between 44° to 63.5° E longitudes. Figure 1 illustrates the location of Iran in the world. Iran is located in an arid and semi-arid climate and this vast country is very diverse in terms of topography and climate. Iran is located in an arid and semi-arid climate and this vast country is very diverse in terms of topography and climate.

#### Data collection and statistical analysis

In this study the data related to the frequency of individuals diagnosed with breast cancer from 2010 to 2014 were used. Information was related to 334 cities of 31 provinces in Iran extracted from Iran Cancer Atlas. The studied data are from breast cancer data at the detection stage.

A generalized estimating equation based method for zero-inflated negative binomial regression model (GEE.ZINB) [14] was used to investigate the effect of geographical longitude and latitude, the angle of radiation and the intensity of radiation during the first and the second six months of the year. The radiation angle is calculated based on latitude. This method takes into account the correlation between cities nested in the provinces.

### Results and discussion

Using data analysis and model used, the following results were obtained, which were summarized in Table 2.

**Table 2:** Results of data analysis based on the model used

Variable	Count part			Zero part		
	Coefficient	SE	P value	Coefficient	SE	P value
Intercept	0.009	0.005	0.087	− 0.002	0.005	0.730
First six month	0.024	0.010	0.018	− 0.011	0.012	0.378
Second six month	0.010	0.006	0.120	− 0.001	0.005	0.858
First six month	0.131	0.059	0.027	− 0.102	0.063	0.104
Second six month	− 0.286	0.098	0.003	0.100	0.098	0.308
Latitude						
Q1	Ref*	SE	P value	coefficient	SE	P value
Q2	− 0.203	0.200	0.309	0.261	0.262	0.319
Q3	− 0.520	0.249	0.037	− 0.172	0.278	0.535
Q4	− 1.047	0.323	0.001	− 0.576	0.314	0.067
Q5	− 1.183	0.353	0.001	− 0.390	0.405	0.336
Longitude						
Q1	Ref*	SE	P value	Coefficient	SE	P value
Q2	− 0.048	0.064	0.447	0.040	0.035	0.253
Q3	0.389	0.077	< 0.001	− 0.407	0.283	0.150

* Reference category

Radiation angle and radiation intensity had a significant effect on the incidence of breast cancer, so that with increasing radiation angle and radiation intensity, the incidence of cancer has increased. Increasing the day length in the first six months of the year has significantly increased the incidence of breast cancer. Longitude was divided into three parts using mean and standard deviation. The higher the latitudes, the significantly higher the incidence of cancer.

Spatial variations in the amount of sunlight caused many differences in the environment and this can affect all aspects of human life. In addition to spatial variations, temporal changes in the amount of solar radiation in different places can have many effects on humans and their health. The amount of solar radiation varies in different parts of the world. The vast country of Iran is located at latitude 25 to 40 degrees north and longitude 44 to 63 degrees east, and due to differences in latitude, there are differences in receiving solar energy. Iran is located in the radiant region of the earth. Ultraviolet radiation in most days of the year is above the standard and although the incidence of cancer is affected by various economic, cultural and environmental (geographical) factors, but among environmental factors the amount of energy receiving from the sun is directly related to the risk of cancer. Ultraviolet radiation that reaches the earth's surface originates from the sun and passes through the earth's atmosphere, during which many absorption and scattering processes occur. Outside the tropics, most radiation occurs when the sun is at its highest point, which is usually about noon (solar noon). Latitude is an effective factor in radiation, and the lower the latitude, the higher the intensity of ultraviolet radiation. The highest intensity of ultraviolet radiation is in summer, which is due to more vertical sunlight in this season.

In order to investigate the effect of sunlight, its intensity and duration on the incidence of breast cancer in Iran, using the relationship between the angle of radiation and latitude, its values were calculated and based on the statistical model its effect on breast cancer was determined.

The findings showed that the angle of radiation and its intensity is directly related to the incidence of breast cancer. Based on the results, the rate of breast cancer in low latitudes is higher than high latitudes. The maximum amount of solar radiation occurs around the orbit of Cancer and this region covers parts of southern Iran. In the southern latitudes of Iran, due to the vertical radiation angle, the amount of radiation intensity is higher than the northern latitudes, but in summer in the northern latitudes, the day length is longer than in the southern areas. More radiation with less intensity is one of the climatic characteristics of the northern latitudes of Iran in summer and in the southern latitudes, the length of the day is shorter but the intensity of radiation is higher. According to the research finding, the higher the radiation intensity at lower latitudes, the significantly higher the rate of breast cancer. Therefore, it can be said that less radiation with higher intensity at low latitudes increases the risk of breast cancer compared to northern latitudes (longer radiation with less intensity). An interesting point that can be seen from the results of this study is that with increasing longitude, the rate of cancer has also increased significantly.

### Discussion and conclusion

Iran is a vast country with a variety of climatic conditions. It is located in a relatively warm region of the world, and many people are constantly exposed to intense sunlight. Therefore, it can be said that environmental factors in Iran cause malignant diseases such as cancer. This study investigated the effect of geographical and climatic factors, intensity of sunlight and daylight on breast cancer in Iran. The findings showed that shorter exposure times with higher intensity at low latitudes increased the risk of breast cancer. On the other hand, the northern latitudes of Iran, since they are affected by rainfall systems and are located in the subtropical region their humidity is higher than southern latitudes. This reflects more of the sun's short waves during the day, and to some extent it can prevent the deadly waves of the sun from entering the earth's surface and affect human health and causes some diseases such as cancer. The factor that really reduces UV radiation is cloud cover.

The results showed that with increasing longitude, the rate of cancer also increases significantly. Since the great deserts of Iran such as Lut plain are located in the eastern regions and the lowlands of the Iranian plateau are located in the eastern and southeastern regions, it can be said that due to the high thickness of the atmosphere in these regions, the amount of wave received by the sun in the electromagnetic spectrum remains mostly on the earth. On the other hand, due to the lack of water vapor, short waves reach the surface of the earth more and it seems that this natural factor has led to the prevalence of breast cancer in the middle lowlands and low altitude areas in the east and southeast of Iran.

In general, sunlight is one of the most potential carcinogens. The southern latitudes of Iran always receive more radiant energy from the sun due to their location around the orbit of Cancer, as well as the vastness of deserts and dry air, and having a more vertical angle of radiation. In these areas, almost all times of the year, especially in summer, because of high altitude of the tropics and clear skies, and also due to vertical radiation of the sun in the orbit of Cancer, the intensity of radiation is very high and this can increase ultraviolet radiation, which stops the immune system and disrupts DNA.

Comparing the obtained results with the studies of others [7, 13, 17, 22], it was found that in Iran, increasing solar radiation in different geographical areas increases the risk of breast cancer. Their studies at higher latitudes considering other environmental parameters by using various methods displayed different results.

## Limitations

One of the main limitations of this study was the lack of long-term and sufficient data on breast cancer in Iran.

## Data Availability

According to the memorandum No. 32/1621 dated 19/05/2020 (through Sayyed Jamaleddin Asadabadi University); the data has been taken from the Cancer Atlas and analyzed. The data is available upon the request from the authors.

## Abbreviations

GEE: Generalized estimating equations
ZINB: Zero-inflated negative binomial
